# Hypertrophic Pyloric Stenosis developing In a Patient Operated for Patent Urachus – A Case Report

**Published:** 2014-05-21

**Authors:** Manoj Kumar Jangid, Yousuf Aziz Khan, Sunil Kumar Yadav, Esmaeel Taqi

**Affiliations:** Department of Paediatric Surgery, Ibn Sina Specialized Surgical Hospital, Al-Sabah Health Region, State of Kuwait.

**Keywords:** Patent urachus, Hypertrophic pyloric stenosis, Neonate

## Abstract

A neonate with patent urachus (PU) who later developed hypertrophic pyloric stenosis (HPS) is being reported. The newborn was first operated for PU; post-operatively he developed persistent vomiting and radiological workup confirmed HPS. Pyloromyotomy was performed with an uneventful recovery.

## INTRODUCTION

Hypertrophic pyloric stenosis (HPS) is a commonly encountered condition in newborns. [1] Associated congenital anomalies may be present in 6–20% of the patients with HPS.[2] The various anomalies reported include those of the central nervous, gastrointestinal, cardiovascular and genitourinary systems.[3] Patent urachus (PU) in a patient who later developed HPS is not reported earlier.

## CASE REPORT

A 19-day-old male newborn was referred to us with a history of clear discharge through the umbilicus. The baby was noted to have clear watery discharge from umbilicus within the first week of life and was treated at a local clinic. By the end of third week, he developed redness around umbilicus. There were complaints of some vomiting after feeds. Clinically the baby was afebrile, active and alert. Abdominal examination showed periumbilical rashes and a clear watery discharge through the umbilicus. No orifice could be seen at the umbilical site.

Ultrasound (US) abdomen showed normal pylorus and bilateral grade I hydronephrosis. An initial impression of a discharging sinus was made clinically and surgical exploration was decided. Surgical exploration was done through the skin crease incision at the lower aspect of umbilical fold. Patent urachus found which was dissected up to the urinary bladder and excised with repair of bladder defect (Fig.1). On the 2nd post-operative day baby developed non-bilious projectile vomiting soon after the feed were started. The X-ray abdomen showed a gas filled distended stomach. US abdomen was repeated, which showed the pyloric length and thickness of 19 mm and 2mm and, 23 mm and 2.5 mm respectively when repeated. Upper GI contrast study was performed because of equivocal US findings which revealed distended stomach with elongated and narrowed pylorus suggestive of HPS (Fig.2). The baby underwent open exploration again on 8th post-operative day of the first surgery, through the circumferential extension of the previous incision over the superior aspect of umbilicus. Findings were confirmed and Ramstedt’s pyloromyotomy was performed. Post-operative course was uneventful.

**Figure:**
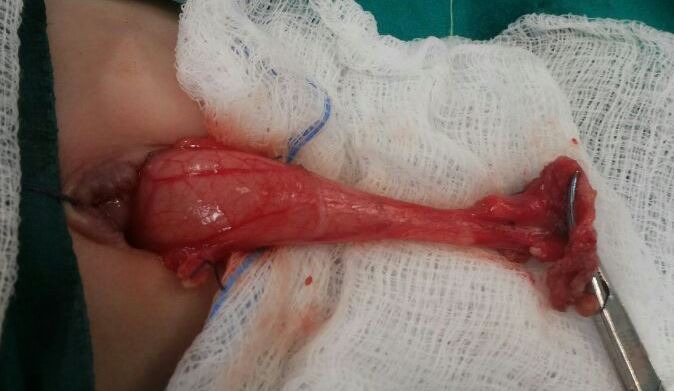
Figure 1:Per-operative picture showing patent urachus extending up to the urinary bladder.

**Figure:**
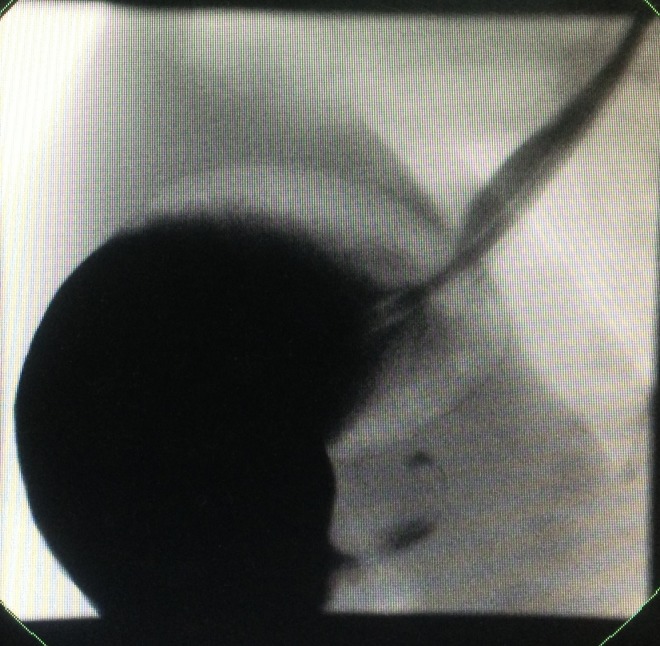
Figure 2:Upper GI contrast study – showing dilated stomach with only some contrast passing through hypertrophied pylorus.

## DISCUSSION

Hypertrophic pyloric stenosis (HPS) is one of common surgical causes of vomiting during infancy and typically presents with a history of non-bilious projectile vomiting during 4th to 6th week of life.[4] A patent urachus (PU) is a rare disorder with a variable incidence.[5] Affected newborns usually present with a continuous or intermittent clear discharge from the umbilicus and periumbilical rash. Similar was the presentation of PU in our case. The presentation of HPS in our case was an unusual. Although he had occasional vomiting before operating upon for PU but ultrasound (US) abdomen did not reveal hypertrophied pylorus. The only possible explanation to this could be the progressive development of pyloric hypertrophy. Similar was the observation by Keckler et al in their study where initial US abdomen which was negative for HPS later on was found to be positive.[6]

## Footnotes

**Source of Support:** Nil

**Conflict of Interest:** None declared

